# A Comparative Study of Birdcage RF Coil Configurations for Ultra-High Field Magnetic Resonance Imaging

**DOI:** 10.3390/s22051741

**Published:** 2022-02-23

**Authors:** Jeung-Hoon Seo, Yeji Han, Jun-Young Chung

**Affiliations:** 1 Neuroscience Research Institute, Gachon University, Incheon 21988, Korea; jeunghoon.seo@gachon.ac.kr; 2 Department of Biomedical Engineering, Gachon University, Incheon 21936, Korea; 3 Department of Neuroscience, College of Medicine, Gachon University, Incheon 21565, Korea

**Keywords:** inductively coupled wireless element, high permittivity material, FDTD method, birdcage RF coil, 7.0 T MRI

## Abstract

Improvements in transmission and reception sensitivities of radiofrequency (RF) coils used in ultra-high field (UHF) magnetic resonance imaging (MRI) are needed to reduce specific absorption rates (SAR) and RF power deposition, albeit without applying high-power RF. Here, we propose a method to simultaneously improve transmission efficiency and reception sensitivity of a band-pass birdcage RF coil (BP-BC RF coil) by combining a multi-channel wireless RF element (MCWE) with a high permittivity material (HPM) in a 7.0 T MRI. Electromagnetic field (EM-field) simulations, performed using two types of phantoms, viz., a cylindrical phantom filled with oil and a human head model, were used to compare the effects of MCWE and HPM on BP-BC RF coils. EM-fields were calculated using the finite difference time-domain (FDTD) method and analyzed using Matlab software. Next, to improve RF transmission efficiency, we compared two HPM structures, namely, a hollow cylinder shape HPM (*hc*HPM) and segmented cylinder shape HPM (*sc*HPM). The *sc*HPM and MCWE model comprised 16 elements (16-rad BP-BC RF coil) and this coil configuration demonstrated superior RF transmission efficiency and reception sensitivity along with an acceptable SAR. We expect wider clinical application of this combination in 7.0 T MRIs, which were recently approved by the United States Food and Drug Administration.

## 1. Introduction

The recent steady increase in the main magnetic field ($B_{0}$-field) strength in magnetic resonance imaging (MRI) systems has been able to improve the signal-to-noise ratio (SNR), among other advantages. Specifically, the 7.0 T MRI, which has recently been approved by the United States Food and Drug Administration (US FDA), offers multiple clinical benefits over the currently used 3.0T MRI [1,2,3]. As improving the performance of radiofrequency (RF) coils is essential for effectively translating the benefits of ultra-high field (UHF) MRI to clinically relevant data, RF coil configurations have played an important role in acquiring UHF MRI. However, as UHF MRIs began to be used in clinical trials and the $B_{0}$-field strength increased, magnetic flux field ($B_{1}$-field) sensitivity and the uniformity of RF coils became crucial design factors of RF coils [4,5,6]. 

Even though the 7.0 T MRI has recently provided limited approval by the US FDA for use in head and extremities (arms and legs) imaging, whole body imaging is not permitted due to safety issues, including tissue heating, which occurs because UHF MRI requires a large amount of RF energy for human imaging [7]. Hence, RF safety studies have evaluated higher specific absorption rates (SAR), which indirectly measure tissue heating upon RF exposure [8,9,10,11,12,13,14,15], and research has focused on signal strength enhancement and SAR safety for RF coils used in 7.0 T MRI approved for clinical use.

The required RF power to excite a spin system increases with the $B_{0}$-field strength. $B_{1}$-field sensitivity is of particular concern in UHF MRI because, as the magnetic field increases, a greater RF frequency is needed to excite the proton (^1^H) nuclei in the human body using an RF pulse, which is critical for high signal sensitivity of the RF coil [16,17,18,19]. Additionally, an insufficient input RF power level causes RF power deposition [20,21,22,23,24], which leads to problems in the SAR. To address this low RF power issue, RF coils that are more sensitive to RF transmission ($B_{1}^{+}$-field) and reception ($B_{1}^{-}$-field) are needed. Further, as such high sensitive coils can provide a higher SNR, along with safety in terms of the SAR, they are recognized as a critical factor for maximizing $B_{1}$-field sensitivity, and hence, studies have actively sought to enhance the signal sensitivity of RF coils [25,26,27,28,29,30,31]. Moreover, research is also focused on using various supplementary coils or materials in conventional coils, apart from inductively coupled wireless elements, to obtain additional $B_{1}$-field signal sensitivity [32,33,34,35,36,37] and various high dielectric materials to improve RF transmission efficiency [38,39,40,41,42]. 

We have previously described a set of electromagnetic field (EM-field) simulations that combine various methods to improve $B_{1}^{+}$-field efficiency and $B_{1}^{-}$-field sensitivity and have proposed the use of an optimal combination of a band-pass type bird cage coil (BP-BC RF coil) with a multi-channel wireless RF element (MCWE) [43]. A BP-BC RF coil was the optimal choice because it provided the greatest uniformity and signal sensitivity among the three types of BC RF coils (Low-pass, High-pass, and Band-pass) tested in UHF MRI [44,45,46]. Although the role of MCWE in improving $B_{1}$-field sensitivity has been evaluated in previous studies, no studies have compared MCWE with optimized combinations of RF coils and additional methods of simultaneously improving $B_{1}^{+}$-field efficiency and $B_{1}^{-}$-field sensitivity. As the B_0_-field strength increases, it is necessary to minimize the SAR as much as possible by minimizing the input RF power level and RF power deposition. To improve the insufficient input RF power level and RF power deposition in a BP-BC RF coil and enable stable performance, an MCWE consisting of 16-elements and high-permittivity material (HPM) was used to simultaneously improve RF transmission and reception.

Analyses of RF transmission in RF coils made with HPM, and of RF reception by various types of dedicated-shape RF coils, have been performed independently. Under certain conditions, depending on the shape of the HPM and the attachment positions, the relative permittivity may be too high, resulting in an inhomogeneity in the $B_{1}^{+}$-field. Given these problems, research related to the optimization of HPM pads is also underway [46]. To ensure optimal HPM performance, both thickness and appropriate structure should be considered, which would depend on the HPM width and the direction of RF wave propagation. Furthermore, HPM application methods have been typically used on RF coils with arbitrary shapes, but HPM with symmetric features is yet to be applied to RF coils to obtain a more homogeneous RF field distribution. 

Thus, this study aimed to identify and propose the use of an optimal combination by comparing and analyzing combinations of MCWE along with shape-optimized HPM for improving $B_{1}^{+}$-field efficiency. The performance of each RF coil combination was compared using EM-field analysis of |$B_{1}$|-, |$B_{1}^{+}$|-, |$B_{1}^{-}$|-, and E-fields and the SAR.

## 2. Materials and Methods

The dimensions of the MCWE were 280 mm × 150 mm (diameter × length) and it consisted of 16-elements, each of size 40 mm × 150 mm, that were placed between the legs of the BP-BC RF coil. Two types of HPM structures were compared, namely, a hollow cylinder shape (*hc*HPM) and a segmented cylinder shape (*sc*HPM). The *hc*HPM consisted of a hollow cylinder with an outer diameter of 315 mm, an inner diameter of 295 mm, and length 150 mm. The dimensions of *sc*HPM were identical to the *hc*HPM but it was divided into 16-elements of the same size as that of MCWE. The dielectric properties of HPM were as follows: relative permittivity = 300 and loss tangent = 0.05. The width of each RF coil element and the *sc*HPM was set to 40 mm so that the MCWE could be placed between the legs of the BP-BC RF coil. The diameter of the MCWE was set to 280 mm to maximize the strength of the induced coupling signal by placing it as close to the human head as possible. The diameter of the BP-BC RF coil was 330 mm, which was the minimum size required for placing the HPM and acrylic formers between the MCWE and the BP-BC RF coil. HPM thickness was set at 20 mm to ensure the maximum efficiency in the limited space between MCWE and BP-BC RF coils. These structures were modeled to be used in the 7.0 T MRI system and the distance between MCWE, HPM, and BP-BC RF coils was set to 10 mm, based on the thickness of commonly used acrylic frames. 

The effects of MCWE were analyzed with HPM, either *hc*HPM or *sc*HPM, located between BP-BC RF coil and MCWE. Specifically, the three different types of structures, i.e., the loop of BP-BC RF coil for leg current, single element of MCWE, and single element of *hc*HPM, were aligned in the same direction. For RF transmission and reception (Tx/Rx), the BC RF coil was used as the main RF coil with a band-pass type structural design. The BP-BC RF coil was operated in the transceiver mode and provided uniform RF to the conductor using current sources (1 A) with a sinusoidal RF pulse. The current RF sources were used under conditions that assumed an ideal current distribution and neglect inter element coupling [47,48,49,50,51,52,53,54,55]. The BP-BC RF coil consisted of 16-legs with 48 current RF sources. The MCWE was tuned to 300 MHz using a tuning capacitor (6.26 pF) and was configured to use only passive elements without RF sources. 

For simulation under ideal conditions, the BP-BC RF coil and MCWE were made from perfect electric conductor (PEC) materials. The *sc*HPM was located between the legs of the BP-BC and at the same position as the MCWE, which also consisted of 16-elements. The dimensions of *sc*HPM were defined as the size between the legs of the BP-BC RF coil, which is consistent with the size of the MCWE. The $B_{1}$-field distribution of BP-BC RF coil is generated in the vertical direction of the closed loop between the legs. If the dimensions of *sc*HPM exceed the size between the legs of the BP-BC RF coil, RF waves can be distorted in the horizontal direction to reduce signal efficiency, so it has been set to a size that can minimize the RF wave distortion. 

For performance evaluation of the BP-BC RF coil, MCWE, and HPM combinations, EM-field simulation models were designed and assessed using xFDTD simulation software (v 6.6) (Remcom, Inc., State College, PA, USA) using the FDTD method based on Yee cells [56]. To evaluate the configurations used in EM-field simulation, we used two types of numerical phantoms: an oil-based cylindrical phantom and a human head model. For relative comparison of EM-field sensitivity and uniformity, we used an oil-based cylindrical phantom with a diameter of 224 mm and a length of 150 mm. For quantitative evaluation of each configuration in the human body, we performed the EM field simulation using a human head model (HIFI head model with 17 tissues properties, provided by Remcom, Inc). The conductivity and relative permittivity of the oil-based cylindrical phantom were 0 S/m and 4, respectively. EM-field simulations were performed with 1 mm^3^ voxel resolution. The oil-based cylindrical phantom simulation performed FDTD operations for 372 × 372 × 190 cells, totaling 26,292,960 voxels, while that for the human phantom simulation was 522 × 372 × 340 cells, totaling 66,022,560 voxels.

For computational analysis, EM-field distributions were analyzed in terms of SAR and |$B_{1}$|, |$B_{1}^{+}$|, |$B_{1}^{-}$|, and |E| fields. To evaluate the effect of HPM and MCWE on EM-fields provided by BP-BC RF coil, EM-field sensitivities were compared for |$B_{1}$|, |$B_{1}^{+}$|, |$B_{1}^{-}$|and |E|-fields. The |$B_{1}^{+}$|-field was used to verify the effectiveness of the HPM, and the |$B_{1}^{-}$|-field was used to verify the effectiveness of the MCWE. The |$B_{1}$|-field was used to evaluate the effect of using HPM and MCWE at the same time. 

The $B_{1}$ contains two circularly polarized components, defined as $B_{1}^{+}$ and $B_{1}^{-}$. Here, $B_{1x}$ and $B_{1y}\;$ are the $B_{1}$ components (same as in magnetization) of the x- and y-axes, respectively. $B_{1}^{+}$ and $B_{1}^{-}$ can be defined as follows:

$$B_{1}^{+}=\left|\frac{\left(B_{x}+iB_{y}\right)}{\sqrt{2}}\;\right|,\;\;B_{1}^{-}=\left|\frac{\left(B_{x}-iB_{y}\right)}{\sqrt{2}}\;\right|$$

A 90° flip-angle with a 3-ms rectangle RF pulse was assumed to be applied to the center of the BP-BC RF coil and the target subject, and the normalized coefficient (Norm-COEF) was calculated based on the assumption that the signal sensitivity of the central voxel in the acquired |$B_{1}^{+}$|-field was 1.95 μT [57,58]. The normalized coefficient was calculated by applying the $B_{1}^{+}$ transmission efficiency (1.95 μT) obtained using the Fast Low-Angle Shot (FLASH) sequence in the actual 7.0 T MRI experiment to the calculated |$B_{1}^{+}$|-field. The FLASH $B_{1}^{+}$ flip angle mapping sequence used in the actual 7.0 T MRI experiment were TE = 1.83 ms, TR = 5000 ms, resolution = 1.95 × 1.95 × 8 mm^3^, flip angle = 85°, rectangular pre-saturation pulse duration = 1.5 ms. The calculated Norm-COEF values were applied to the SAR maps for quantitative SAR analysis, which was performed under conditions identical to those of the actual MRI experiment by applying Norm-COEF values to the SAR map. To assess the safety of UHF MRI, the SAR analysis was performed in terms of RF safety. The SAR maps were evaluated using the whole-averaged SAR (mean SAR) and Max SAR values. The whole-averaged SAR value was calculated using the mean value of un-averaged SAR map and Max SAR value was calculated using maximum values of un-averaged SAR map.

## 3. Results & Discussion

**Figure 1:**
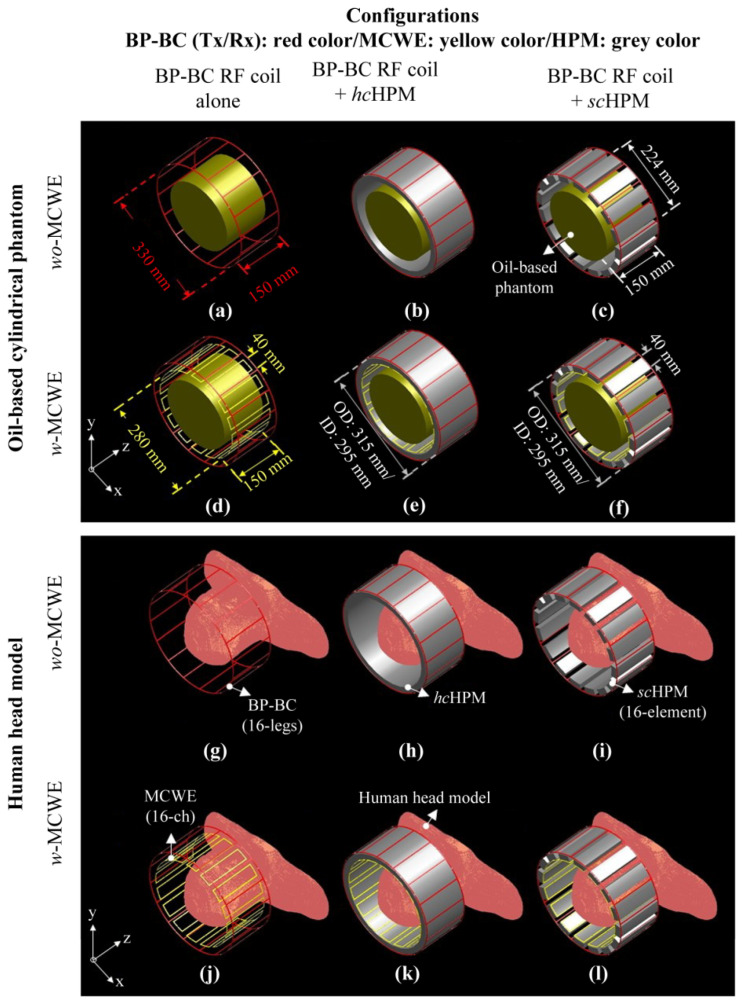
Configurations of BP-BC, MCWE, and HPM for EM-field simulations using oil-based cylindrical phantom (**a**–**f**) or human body model (**g**–**l**): (**a**,**g**) BP-BC RF coil alone − *wo*-MCWE; (**b**,**h**) BP-BC RF coil + *hc*HPM − *wo*-MCWE; (**c**,**i**) BP-BC RF coil + *sc*HPM − *wo*-MCWE; (**d**,**j**) BP-BC RF coil alone − *w*-MCWE; (**e**,**k**) BP-BC RF coil + *hc*HPM − *w*-MCWE; (**f**,**l**) BP-BC RF coil + *sc*HPM − *w*-MCWE.

To verify the effect of HPM on EM-fields generated by the BP-BC RF coil, we performed EM-field simulations (Figure 1) using three designs, i.e., a BP-BC RF coil, a BP-BC RF coil with an *hc*HPM, and a BP-BC RF coil with an *sc*HPM. Next, to evaluate the impact of simultaneous use of an MCWE and HPM, we compared the above configurations of BP-BC with an MCWE (*w*-MCWE) and without an MCWE (*wo*-MCWE). Figure 1 depicts the design of the numerical EM-field simulation models. Thus, the EM-field simulation models for the oil-based cylindrical phantom for a *wo*-MCWE were the BP-BC RF coil alone (BP-BC RF coil alone − *wo*-MCWE) (Figure 1a), the BP-BC RF coil with an *hc*HPM (BP-BC RF coil + *hc*HPM − *wo*-MCWE) (Figure 1b), and the BP-BC RF coil with an *sc*HPM (BP-BC RF coil + *sc*HPM − *wo*-MCWE) (Figure 1c), while those for the *w*-MCWE condition were the BP-BC RF coil alone (BP-BC RF coil alone − *w*-MCWE) (Figure 1d), the BP-BC RF coil with an *hc*HPM insertion (BP-BC RF coil + *hc*HPM − *w*-MCWE) (Figure 1e), and the BP-BC RF coil with an *sc*HPM insertion (BP-BC RF coil + *sc*HPM − *w*-MCWE) (Figure 1f). The *wo*-MCWE and *w*-MCWE conditions for the human head model were similarly designed as the BP-BC RF coil alone − *wo*-MCWE (Figure 1g), the BP-BC RF coil + *hc*HPM − *wo*-MCWE (Figure 1h), and the BP-BC RF coil + *sc*HPM − *wo*-MCWE (Figure 1i) for the *wo*-MCWE condition, and as the BP-BC RF coil alone − *w*-MCWE (Figure 1j), the BP-BC RF coil + *hc*HPM − *w*-MCWE (Figure 1k), and the BP-BC RF coil + *sc*HPM − *w*-MCWE (Figure 1l) for the *w*-MCWE condition. 

**Figure 2:**
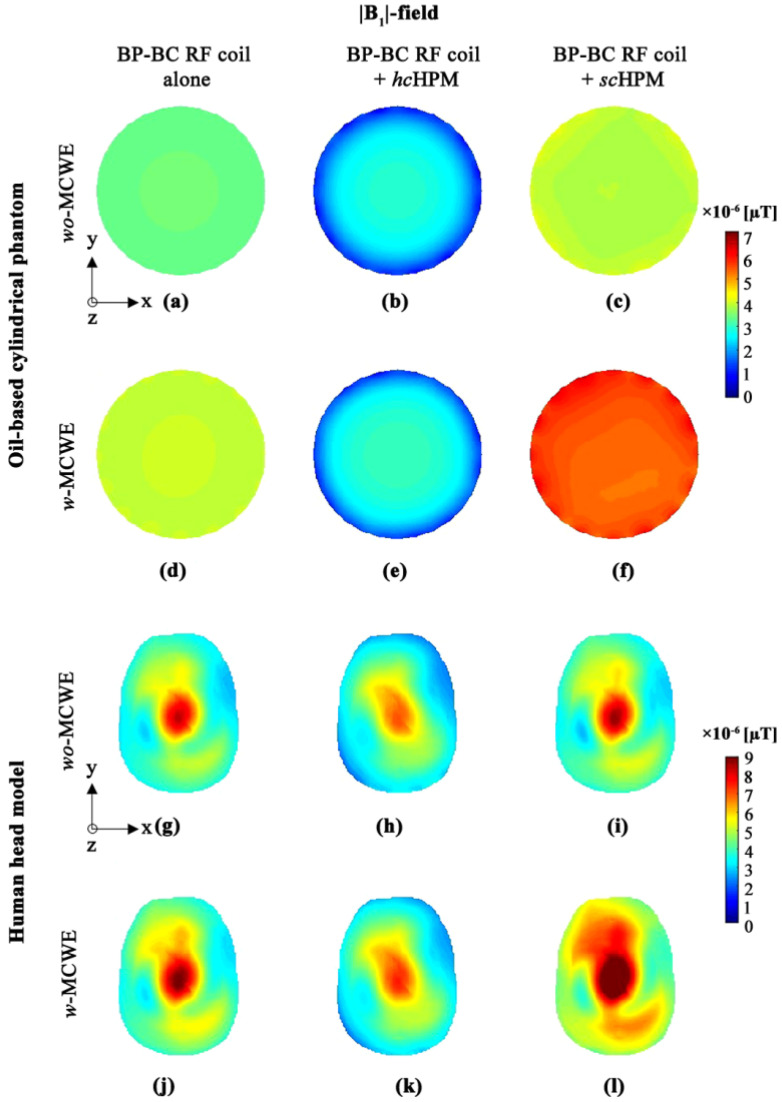
|$B_{1}$|-field distribution in oil-based cylindrical phantom (**a**–**f**) or human body model (**g**–**l**): (**a**,**g**) BP-BC RF coil alone − *wo*-MCWE; (**b**,**h**) BP-BC RF coil + *hc*HPM − *wo*-MCWE; (**c**,**i**) BP-BC RF coil + *sc*HPM − *wo*-MCWE; (**d**,**j**) BP-BC RF coil alone − *w*-MCWE; (**e**,**k**) BP-BC RF coil + *hc*HPM − *w*-MCWE; (**f**,**l**) BP-BC RF coil + *sc*HPM − *w*-MCWE.

The EM-field was numerically calculated and its value at the center slice in the |$B_{0}$|-direction (along z-axis) of the BP-BC RF coil was used for comparison. The |$B_{1}$|-field results for the various configurations of the BP-BC RF coil were computed for the oil-based cylindrical phantom and a human head model, as shown in Figure 2. For RF transmission and reception, we derived and compared values for the |$B_{1}$|-, |$B_{1}^{+}$|-, and |$B_{1}^{-}$|-fields. Detailed values for the |$B_{1}$|-, |$B_{1}^{+}$|-, and |$B_{1}^{-}$|-field sensitivity and standard deviation (SD) are listed in Table 1. 

The |$B_{1}$|-field results for the various configurations of the BP-BC RF coil (Figure 2) were compared based on signal sensitivity, and |$B_{1}$|-field sensitivity at the center-point of the BP-BC RF coil was used for qualitative comparison. In the oil-based cylindrical phantom, |$B_{1}$d, as shown in Figure 2a,d, and the *wo-*MCWE condition produced characteristic high signal sensitivity and uniformity. However, higher |$B_{1}$|-field sensitivity was observed in the *w*-MCWE condition, indicating that the MCWE improves |$B_{1}$|-field sensitivity. To analyze the effects of the two types of HPM, |$B_{1}$|-fields for the *hc*HPM (Figure 2b,e) and *sc*HPM (Figure 2c,f) were compared in the *wo*-MCWE and *w*-MCWE conditions. For the *hc*HPM in the *wo*-MCWE and *w*-MCWE conditions, performance degradation occurred in the |$B_{1}$|-field, characterized by rapid signal reduction in the periphery. Specifically, compared to the BP-BC RF coil alone, |$B_{1}$|-field sensitivity was reduced by approximately 16.217% and 24.415% under the *wo*-MCWE (Figure 2b) and *w*-MCWE (Figure 2e) conditions, respectively. However, with the *sc*HPM, higher |$B_{1}$|-field sensitivity than the BP-BC RF coil alone was observed. Next, a combination of the *sc*HPM and *wo*-MCWE (Figure 2c) improved |$B_{1}$|-field sensitivity by approximately 9.742% compared to the BP-BC RF coil alone. Notably, |$B_{1}$|-field sensitivity dramatically improved by about 32.130% in the *sc*HPM and *w*-MCWE configuration compared to the BP-BC RF coil alone (Figure 2f).

As demonstrated in Figure 2, the |$B_{1}$|-field simulation results with the human head model showed greater sensitivity, validating the effectiveness of using both an MCWE and HPM. Figure 2g shows the results for the *wo*-MCWE condition, where the BP-BC RF coil generated a typical |$B_{1}$|-field distribution with high sensitivity and uniformity. As seen in Figure 2j, |$B_{1}$|-field sensitivity was enhanced under *w*-MCWE conditions (Figure 2g) due to the presence of the MCWE. From an architectural perspective, the |$B_{1}$|-field for the human head phantom with an *hc*HPM (Figure 2h,k) exhibited performance degradation under both the *wo*-MCWE and *w*-MCWE conditions compared to the BP-BC RF coil alone condition, i.e., |$B_{1}$|-field sensitivity was reduced by approximately 14.198% in the *wo*-MCWE condition (Figure 2h) and by approximately 15.924% in the *w*-MCWE condition (Figure 2k). In contrast, the |$B_{1}$|-field results using an *sc*HPM showed an improved performance by about 2.542% in the *wo*-MCWE (Figure 2i) condition and about 22.174% in the *w*-MCWE condition (Figure 2l) compared to the BP-BC RF coil alone. 

**Figure 3:**
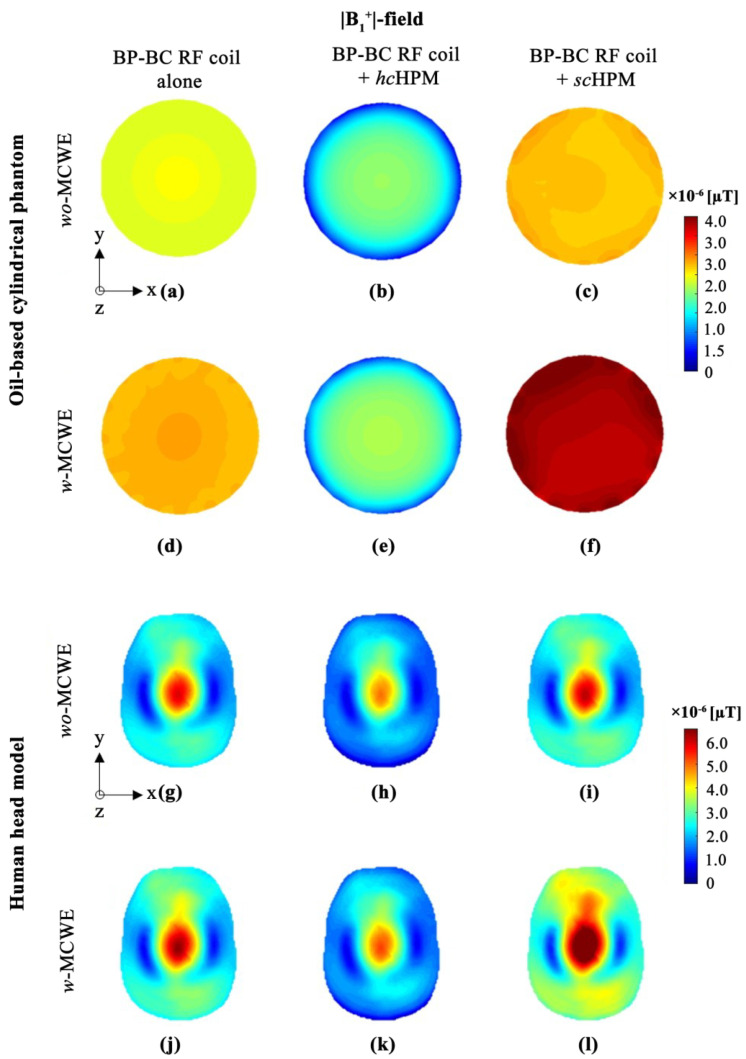
|$B_{1}^{+}$|-field distribution in oil-based cylindrical phantom (**a**–**f**) or human body model (**g**–**l**): (**a**,**g**) BP-BC RF coil alone − *wo*-MCWE; (**b**,**h**) BP-BC RF coil + *hc*HPM − *wo*-MCWE; (**c**,**i**) BP-BC RF coil + *sc*HPM − *wo*-MCWE; (**d**,**j**) BP-BC RF coil alone − *w*-MCWE; (**e**,**k**) BP-BC RF coil + *hc*HPM − *w*-MCWE; (**f**,**l**) BP-BC RF coil + *sc*HPM − *w*-MCWE.

As the |$B_{1}$|-fields were computed using the |$B_{1}^{+}$|- and |$B_{1}^{-}$|-fields according to the reciprocity theorem, analysis of the |$B_{1}^{+}$|- and |$B_{1}^{-}$|-fields was deemed essential for quantitative analysis of the |$B_{1}$|-field results. The signal sensitivities of the |$B_{1}^{+}$|- and |$B_{1}^{-}$|-fields were compared using the calculated maximum values. The |$B_{1}^{+}$|-fields obtained with the oil-based cylindrical phantom and human head model are shown in Figure 3, while Figure 3a,d depict the oil-based cylindrical phantom fields with the BP-BC RF coil under *wo*-MCWE and *w*-MCWE conditions, respectively. In Figure 3a, the maximum value of the |$B_{1}^{+}$|-field generated by the BP-BC RF coil under the *wo*-MCWE condition was calculated to be 0.247 × 10^−5^ μT, whereas that under the *w*-MCWE condition (Figure 3d) was 0.284 × 10^−5^ μT. Under the *w*-MCWE condition (Figure 3d), the |$B_{1}^{+}$|field efficiency was approximately 15.113% better than that under the *wo*-MCWE condition (Figure 3a). When *hc*HPM was used for the oil-based cylindrical phantom (Figure 3b,e), the maximum value of the |$B_{1}^{+}$|-field was calculated to be 0.207 × 10^−5^ μT for the *wo*-MCWE condition (Figure 3b) and 0.215 × 10^−5^ μT for the *w*-MCWE condition (Figure 3e). This corresponds to a 16.207% decrease in the *wo*-MCWE condition and a 24.392% reduction in the *w*-MCWE condition as compared with the BP-BC RF coil alone. In contrast, the BP-BC RF coil with an *sc*HPM insertion improved the |$B_{1}^{+}$|-field efficiency with a maximum value of 0.291 × 10^−5^ μT under the *wo*-MCWE condition (Figure 3c) and 0.443 × 10^−5^ μT under the *w*-MCWE condition (in Figure 3f). Thus, in comparison with the BP-BC RF coil alone, the |$B_{1}^{+}$|-field efficiency improved by approximately 17.868% under the *wo*-MCWE condition and 55.755% under the *w*-MCWE condition with an *sc*HPM. Furthermore, in comparison with *hc*HPM, the |$B_{1}^{+}$|-field efficiency improved by approximately 40.667% under *wo*-MCWE conditions and 106.006% under *w*-MCWE conditions.

For the human head model, the maximum value of the |$B_{1}^{+}$|-field using only the BP-BC RF coil was 0.588 × 10^−5^ μT under the *wo*-MCWE condition (Figure 3g) and 0.637 × 10^−5^ μT under the *w*-MCWE condition (Figure 3j). With an *hc*HPM (Figure 3h,k), this maximum value was 0.491 × 10^−5^ μT under the *wo*-MCWE condition (Figure 3h) and 0.522 × 10^−5^ μT under the *w*-MCWE condition (Figure 3k). With an *sc*HPM (Figure 3i,l), the maximum value was 0.609 × 10^−5^ μT under the *wo*-MCWE condition (Figure 3i) and 0.781 × 10^−5^ μT under the *w*-MCWE condition (Figure 3l).

Next, the use of an *hc*HPM reduced |$B_{1}^{+}$|-field efficiency compared to the BP-BC RF coil alone, and the reduction was calculated to be approximately 16.527% under *wo*-MCWE and 18.071% under *w*-MCWE conditions, respectively. Contrastingly, |$B_{1}^{+}$|-field efficiency improved by 3.604% under *wo*-MCWE conditions and by 22.593% under *w*-MCWE conditions compared to the BP-BC RF coil alone on using an *sc*HPM. With an *sc*HPM, |$B_{1}^{+}$|-field efficiency was greater compared to the BP-BC RF coil + *hc*HPM; specifically, the improvement was calculated to be approximately 24.119% under *wo*-MCWE and 49.635% under *w*-MCWE conditions, respectively.

**Figure 4:**
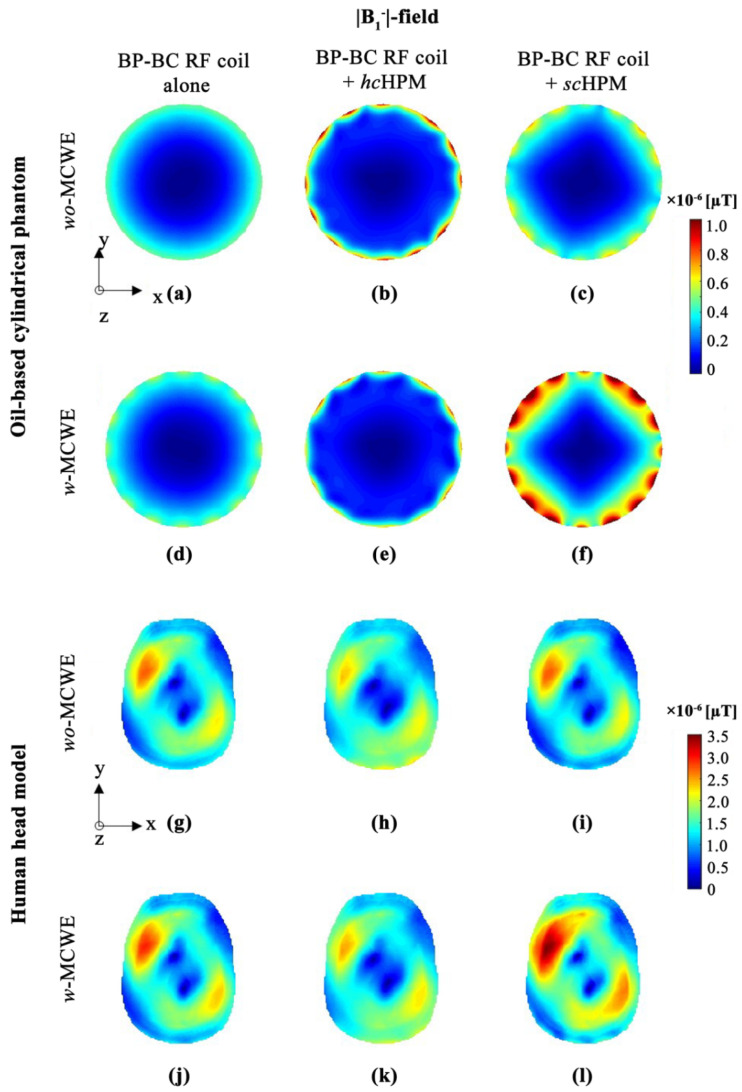
|$B_{1}^{-}$|-field distribution in oil-based cylindrical phantom (**a**–**f**) or human body model (**g**–**l**): (**a**,**g**) BP-BC RF coil alone − *wo*-MCWE; (**b**,**h**) BP-BC RF coil + *hc*HPM − *wo*-MCWE; (**c**,**i**) BP-BC RF coil + *sc*HPM − *wo*-MCWE; (**d**,**j**) BP-BC RF coil alone − *w*-MCWE; (**e**,**k**) BP-BC RF coil + *hc*HPM − *w*-MCWE; (**f**,**l**) BP-BC RF coil + *sc*HPM − *w*-MCWE.

As shown in Figure 4, the signal sensitivity of the |$B_{1}^{-}$|-field, which corresponds to the receiving field, should have zero value according to the reciprocity theorem; however, these values are not so due to inhomogeneity of the |$B_{1}$|-field in the UHF MRI systems. Nevertheless, the signal sensitivity at the center of the |$B_{1}^{-}$|-field has a value close to zero, and a sharp change in |$B_{1}^{-}$|-field sensitivity was identified in the peripheral region of the phantoms used. Therefore, simultaneous analysis of signal sensitivity and SD values for the |$B_{1}^{-}$|-field were performed.

As seen in Figure 4a,d, |$B_{1}^{-}$|-field results for the BP-BC RF coil with the oil-based cylindrical phantom, under *wo*-MCWE (Figure 4a) and *w*-MCWE (Figure 4d) conditions, showed no significant changes in signal sensitivity or uniformity; however, changes in field uniformity were identified at the boundary of the oil-based cylindrical phantom under the *w*-MCWE condition (Figure 4d). For the BP-BC RF coil + *hc*HPM (Figure 4b,e) or BP-BC RF coil + *sc*HPM (Figure 4c,f), abnormal |$B_{1}^{-}$|-field patterns were identified at closed loop positions between the legs of the BP-BC RF coil. These irregular |$B_{1}^{-}$|-field patterns were seen upon HPM and MCWE use as well. In the configuration BP-BC RF coil with MCWE, changes occurred only in |$B_{1}^{-}$|-field patterns, while for the BP-BC RF coil with an HPM and MCWE, changes were seen in both sensitivity and |$B_{1}^{-}$|-field patterns. Specifically, the combination of an *sc*HPM and *w*-MCWE (Figure 4f) allowed us to observe extreme changes in |$B_{1}^{-}$|-field sensitivity in the periphery of the oil-based cylindrical phantom as it was located close enough to the MCWE and the HPM to yield irregular patterns in the peripheral regions. Similar abnormal |$B_{1}^{-}$|-field patterns in the periphery were not observed in the human head model results. 

A comparison of |$B_{1}^{-}$|-field results for the BP-BC RF coil alone for the oil-based cylindrical phantom (Figure 4a,d) and the human head model (Figure 4g,j) showed that values were approximately 2.254~3.506 times higher under the *wo*-MCWE condition (in Figure 4g) and 2.376~3.297 times higher under the *w*-MCWE condition (in Figure 4j) for the human head model. The |$B_{1}^{-}$|-field sensitivity and SD values for the BP-BC RF coil + *hc*HPM for the human head model (Figure 4h,k) were calculated to be relatively low under both *wo*-MCWE and *w*-MCWE conditions. On the other hand, |$B_{1}^{-}$|-field sensitivity and SD distribution for the human head model with the BP-BC RF coil + *sc*HPM under *wo*-MCWE conditions (Figure 4i) did not reveal significant differences from the BP-BC RF coil alone (Figure 4g). The highest |$B_{1}^{-}$|-field sensitivity and SD values were seen with the human head model for the BP-BC RF coil + *sc*HPM under the *w*-MCWE condition (in Figure 4l). 

**Figure 5:**
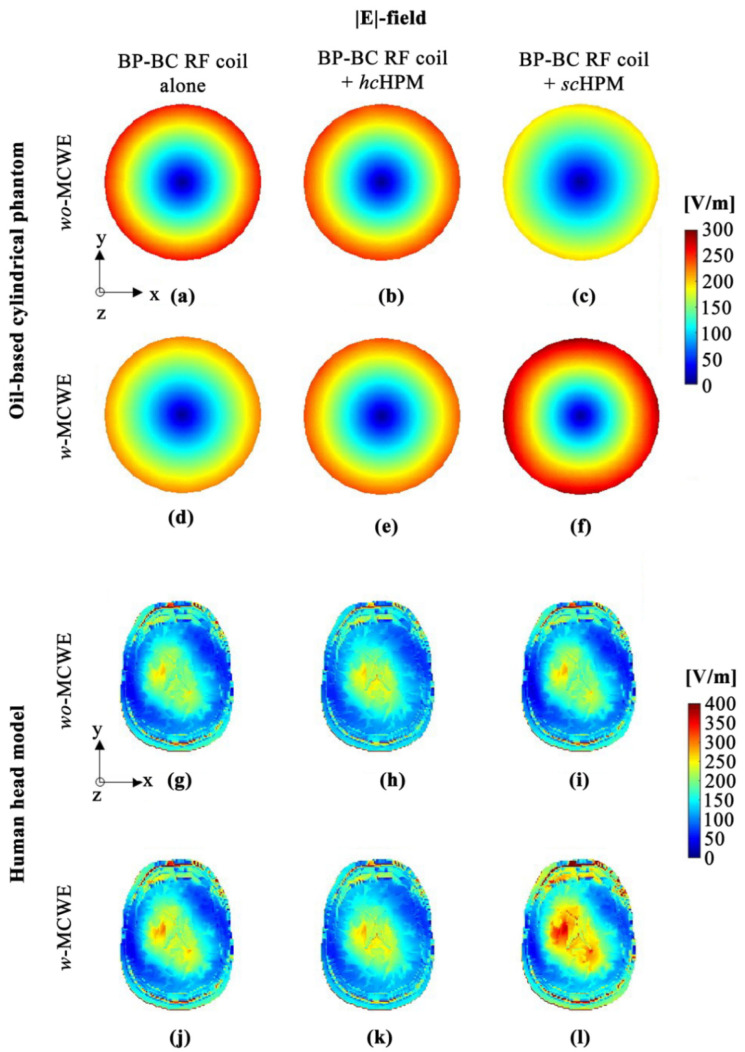
|E|-field distribution in oil-based cylindrical phantom (**a**–**f**) and human body model (**g**–**l**): (**a**,**g**) BP-BC RF coil alone − *wo*-MCWE; (**b**,**h**) BP-BC RF coil + *hc*HPM − *wo*-MCWE; (**c**,**i**) BP-BC RF coil + *sc*HPM − *wo*-MCWE; (**d**,**j**) BP-BC RF coil alone − *w*-MCWE; (**e**,**k**) BP-BC RF coil + *hc*HPM − *w*-MCWE; (**f**,**l**) BP-BC RF coil + *sc*HPM − *w*-MCWE.

As shown in Figure 5 and Table 1, |E|-field maps were analyzed as maximum values and distribution patterns, and the results revealed an |E|-field in the peripheral region of the oil-based cylindrical phantom because this phantom, which was used to control distortion due to EM-field non-uniformity in the UHF, was set to zero conductivity (0 S/m) and a relative permittivity of four. On the other hand, results for the human head model with various dielectric properties show that the |E|-field was at the center, rather than the periphery.

Notably, the oil-based cylindrical phantom results show conflicts between the *wo*-MCWE and *w*-MCWE conditions. Specifically, the maximum value of the |E|-field was calculated with the BP-BC RF coil alone − *wo*-MCWE condition (260.257 V/m; Figure 5a), while the values for the BP-BC RF coil + *hc*HPM − *wo*-MCWE (251.539 V/m; Figure 5b) and BP-BC RF coil + *sc*HPM − *wo*-MCWE (202.869 V/m; Figure 5c) were lower. Under the *w*-MCWE condition, maximum values for the |E|-field were calculated in the order opposite to that seen in the *wo*-MCWE condition, i.e., BP-BC RF coil alone − *w*-MCWE (224.388 V/m; Figure 5d), BP-BC RF coil + *hc*HPM − *w*-MCWE (246.670 V/m; Figure 5e), and BP-BC RF coil with *sc*HPM (296.424 V/m; Figure 5f). Under the *wo*-MCWE condition, |E|-field variance was observed in the periphery due to HPM, but under the *w*-MCWE condition, |E|-field concentration was observed in the periphery due to both HPM and MCWE. 

Unlike that seen with the oil-based cylindrical phantom, the |E|-field was concentrated at the center of the object in the human head model. Under the *wo*-MCWE condition, the maximum value of the |E|-field was calculated at 727.420 V/m for the BP-BC RF coil alone − *wo*-MCWE (Figure 5g), at 567.106 V/m for the BP-BC RF coil + *hc*HPM − *wo*-MCWE (Figure 5h), and at 711.303 V/m for the BP-BC RF coil + *sc*HPM − *wo*-MCWE (Figure 5i). Under the *w*-MCWE condition, the maximum value of the |E|-field was calculated at 740.012 V/m for the BP-BC RF coil alone (Figure 5j), at 586.246 V/m for the BP-BC RF coil alone − *w*-MCWE (Figure 5k), and at 918.380 V/m for the BP-BC RF coil + *hc*HPM − *w*-MCWE (Figure 5l). Thus, the |E|-field results for the oil-based cylindrical phantom and the human head model showed the highest value for the BP-BC RF coil + *sc*HPM − *w*-MCWE (Figure 5f,l). Further, values for the human head model were 24.103~56.654% higher than those of other configurations.

**Figure 6:**
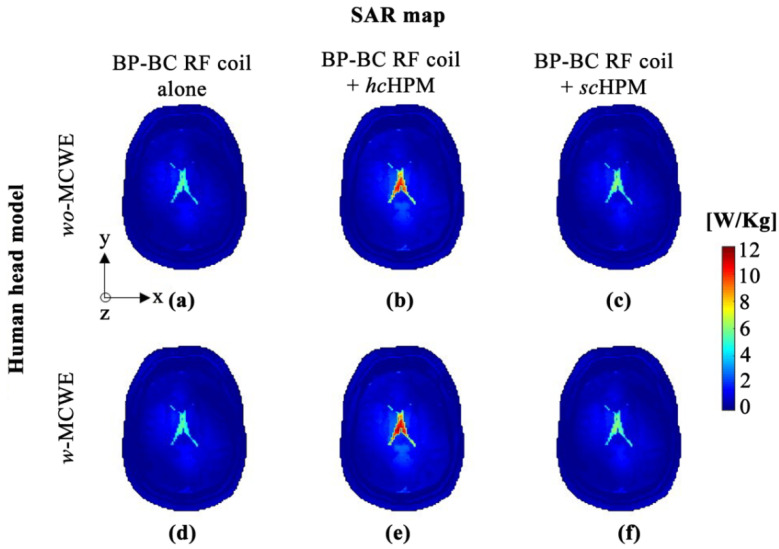
SAR map constructed using Norm-COEF values: (**a**) BP-BC RF coil alone − *wo*-MCWE; (**b**) BP-BC RF coil + *hc*HPM − *wo*-MCWE; (**c**) BP-BC RF coil + *sc*HPM − *wo*-MCWE; (**d**) BP-BC RF coil alone − *w*-MCWE; (**e**) BP-BC RF coil + *hc*HPM − *w*-MCWE; (**f**) BP-BC RF coil + *sc*HPM − *w*-MCWE.

Unlike EM-field analysis results, such as |$B_{1}$|-, |$B_{1}^{+}$|-, |$B_{1}^{-}$|-, and |E|-fields, the SAR map was calculated assuming conditions wherein the coils apply 90° RF, which is similar to real-world MRI. Hence, we used a method to calculate necessary normalized values and then applied the computed values to the SAR map with the |$B_{1}^{+}$|-field subjected to 90° RF. The calculated values were defined by the Norm-COEF, as shown in Table 2, for the BP-BC RF coil alone. The SAR map with the Norm-COEF was calculated and analyzed as shown in Figure 6 and Table 2. The Norm-COEF values under the *wo*-MCWE condition were calculated to be 0.362 for the BP-BC RF coil alone − *wo*-MCWE, 0.431 for the BP-BC RF coil + *hc*HPM − *wo*-MCWE, and 0.352 for the BP-BC RF coil + *sc*HPM − *wo*-MCWE. Under the *w*-MCWE condition, these values were 0.334 for the BP-BC RF coil alone − *w*-MCWE, 0.406 for the BP-BC RF coil + *hc*HPM − *w*-MCWE, and 0.273 for the BP-BC RF coil + *sc*HPM − *w*-MCWE. Norm-COEF values were calculated to be lower for the BP-BC RF coil + *sc*HPM − *w*-MCWE than other configurations due to the former’s high signal sensitivity. 

The quantitative analysis of SAR maps without Norm-COEF values is not possible under ideal conditions, but qualitative SARs were comparable. For the quantitative analysis of SAR maps, we compared whole-averaged SAR (mean SAR) and maximum SAR values. Under the *wo*-MCWE condition, the mean SAR values were calculated to be 0.209 W/Kg for the BP-BC RF coil alone − *wo*-MCWE (Figure 6a), 0.245 W/Kg for the BP-BC RF coil + *hc*HPM − *wo*-MCWE (Figure 6b), and 0.215 W/Kg for the BP-BC RF coil + *sc*HPM − *wo*-MCWE (Figure 6c). Under the *w*-MCWE condition, mean SAR values were calculated to be 0.216 W/Kg for the BP-BC RF coil alone − *w*-MCWE (Figure 6d), 0.235 W/Kg for the BP-BC RF coil + *hc*HPM − *w*-MCWE (Figure 6e), and 0.211 W/Kg for the BP-BC RF coil + *sc*HPM − *w*-MCWE (Figure 6f). No drastic changes in mean SAR values were identified, while extreme changes were observed for maximum SAR values with the BP-BC RF coil + *hc*HPM, which were 19.138~21.358% higher than those for the BP-BC RF coil alone. Significant changes in maximum SAR values were not seen between the BP-BC RF coil + *sc*HPM and the BP-BC RF coil alone under either *wo*-MCWE or *w*-MCWE conditions. However, sharp changes in maximum SAR values were seen with the BP-BC RF coil + *hc*HPM.

EM-field simulation results show that the |$B_{1}$|-field sensitivity of the BP-BC RF coil, combined with MCWE and *sc*HPM, was greater than that of the BP-BC RF coil alone by about 32.130% for the cylindrical phantom and by 22.174% for the human head model. |$B_{1}^{+}$|-field values showed a greater efficiency for the cylindrical phantom (approximately 55.755% improvement) and the human head model (approximately 22.593% improvement). Similarly, the |$B_{1}^{-}$|-field results showed a signal sensitivity improvement of about 122.957% for the cylindrical phantom and about 18.662% for the human head model. Thus, we demonstrate an increase in RF transmission efficiency and reception sensitivity, implying an improvement of RF transmission and reception efficiency, which directly affects |$B_{1}$|-field sensitivity.

Meanwhile, |E|-field results showed a relative increase (32.103% for cylindrical phantom and 24.103% for head models) in the |E|-field concentrations for the configuration of the BP-BC RF coil + *sc*HPM − *w*-MCWE due to the presence of MCWE and *sc*HPM structures. These |E|-field concentration results are expected to have a direct effect on MR images acquired using high-speed RF pulse sequences, such as EPI pulse sequences; however, they did not show significant differences in SAR maps. The combination of MCWE and *sc*HPM provides safety in terms of SAR due to relatively low RF power applied to the BP-BC RF coils. Assuming that actual 90° RF was applied, both the RF transmission and reception efficiency of the BP-BC RF coil + *sc*HPM − *w*-MCWE were increased, resulting in a relatively low RF output. Therefore, the calculated change in SAR values was below 1.993% for the mean SAR and below 1.527% for the maximum SAR value, which are not expected to have a significant impact on human safety.

Here, we describe the results of the EM-field simulations of the BP-BC RF coils with an MCWE and HPM that were optimized to improve RF transmission and reception efficiency. While the BC RF coil has been widely used in MRI because it provides a highly uniform |$B_{1}$|-field distribution, a decrease in RF frequency length after increasing the main magnetic field strength reduces the |$B_{1}$|-field sensitivity, which acts as a critical weakness for volume coils such as BP-BC RF coils. Thus, to improve limited |$B_{1}$|-field sensitivity due to the reduced RF transceiver efficiency in UHF MRI, an optimized configuration with MCWE for improving RF reception efficiency and an HPM for enhancing RF transmission efficiency can be adopted.

We show that RF transmission and reception efficiency can be improved in 7.0 T MRI by enhancing |$B_{1}$|-field sensitivity using a combination of an HPM and MCWE; specifically, that an *sc*HPM improves |$B_{1}^{+}$|-field efficiency of RF transmission, while an MCWE improves |$B_{1}^{-}$|-field sensitivity of RF reception. Further, we show that the shape and arrangement of the HPM are important factors and that the HPM has to be designed in the same direction as the direction of progression of the RF pulse. For example, in the case of the *hc*HPM, a reverse offset of RF waves within the HPM of the hollow cylinder phantom resulted in reduced Tx efficiency. Although RF waves need to be applied perpendicular to the human body, the *hc*HPM distorts RF waves in the horizontal direction, which reduces signal efficiency due to a shielding effect. In contrast, with the *sc*HPM, RF wave distortion was minimized as the HPM elements consisted of separate forms, which only further boosted Tx performance.

In fact, the HPM structures have been mainly used in energy storage technologies such as dielectric-based capacitors, and their main role was to increase energy density. In MRI applications, the HPM should be used in the direction of transmission field propagation to increase the energy density of the transmission field. If the HPM is configured to distort the direction of the transmission field propagation, the energy density of the transmission field decreases, resulting in |$B_{1}^{+}$|-field efficiency decreases.

In this study, since the *hc*HPM structure was configured in a hollow cylinder shape, the transmission field of the BP-BC RF coil was distorted, and the |$B_{1}^{+}$|-field efficiency reduced. On the other hand, the *sc*HPM structure provided the result of improving the |$B_{1}^{+}$|-field efficiency by increasing the energy density without distortion in the direction of the transmission field as HPM structures were configured within the range not exceeding the BP-BC RF coil size.

Furthermore, the synergy between the MCWE and *sc*HPM configurations allowed the |$B_{1}$|-, |$B_{1}^{+}$|- and |$B_{1}^{-}$|-fields in the BP-BC RF coil to dramatically heighten the signal efficiency. Compared to the BP-BC RF coil + *sc*HPM − *wo*-MCWE conditions, the increase in the |$B_{1}$|- and |$B_{1}^{+}$|-field efficiency under *w*-MCWE conditions was found to be much higher due to the use of the *sc*HPM, and the synergy between the *sc*HPM and MCWE was found to be remarkable. On the SAR maps that were constructed using Norm-COEF values, the BP-BC RF coil + *sc*HPM was not significantly different from the BP-BC RF coil alone, while the BP-BC RF coil + *hc*HPM showed both a rapid increase and concentration of SAR in the center of the brain. Importantly, we confirm that the MCWE is significantly involved in overall SAR changes. Even though the superiority of the *sc*HPM was verified by comparing the *hc*HPM and *sc*HPM, only multi-channel forms were considered for wireless coils. Analyzing optimized forms of wireless coils and HPM requires further research on the combination of volumetric wireless coils, such as a birdcage coil and multi-channel RF coil in a phase array, which would function as an RF transceiver. Thus, applying BP-BC RF coils with an MCWE and *sc*HPM to whole-body phantoms takes into account the signal efficiency improvement and SAR reduction, and even though research on other optimized forms of wireless coils and HPM combinations was insufficient, the efficiency of the proposed method is thought to be fully validated. 

Our calculations also show that the proposed combination of the BP-BC RF coil with an MCWE and *sc*HPM displays enhanced |$B_{1}^{-}$|-field sensitivity and |$B_{1}^{+}$|-field efficiency in the human head model. Importantly, SAR values of the proposed combination indicate its suitability and safety for clinical use in humans. 

Our future work aims to evaluate combinations of birdcage-shaped wireless coils and HPM to improve the performance of multi-channel RF coils for the Tx/Rx mode. Further, limited EM-field simulations using a BaTio_3_ material have been performed to verify the effect on HPM, but further evaluation of HPM with varying permittivity and loss parameters is needed.

## 4. Conclusions

In this paper, we describe configurations of BP-BC RF coils that yield improved RF signal sensitivity and safety for use in UHF MRI and propose an optimized combination of a wireless RF coil (for better reception efficiency) and HPM with dielectric properties (for better transmission efficiency). EM-field simulations were performed to verify the effects of the proposed configurations. The BP-BC RF coil, combined with MCWE and *sc*HPM structures, were compared with *hc*HPM configurations, analyzed, and proposed as an optimized configuration based on EM-field simulations. The structure of the *sc*HPM was designed to match the direction of propagation of the RF wave generated by the BP-BC RF coil and the location of both the *sc*HPM and MCWE. Notably, the design and dimensions of both the *sc*HPM and MCWE were such that they could be located between the legs of the BC coil. Further, the optimized structure and the layout of the BP-BC RF coil, combined with the MCWE and *sc*HPM, should be aligned with the direction of the RF field. Both the MCWE and *sc*HPM were designed as a 16-element combination as the RF coil was a 16-leg BP-BC RF coil. 

The optimized combination of MCWE and *sc*HPM improved both the transmission and reception signal efficiency and sensitivity of the BP-BC RF coils in UHF MRI, and we expect its use to improve the sensitivity of birdcage coils that are limited by a low RF power and SAR issues when used in UHF MRIs at or above 7.0 T. Importantly, we believe that the proposed RF coil combination can be effectively applied to MRI systems above 7.0 T. 

One limitation encountered during this study was that the application of the *sc*HPM and MCWE to actual clinical 7.0 T MRI failed to perform comparative studies with electromagnetic field simulation results. MR image acquisition using unauthorized devices is strictly restricted at clinical sites. Due to strict MRI safety regulations for the human patients, actual *sc*HPM and MCWE experiments could not be performed. 

However, the possibility of the *sc*HPM and MCWE has been confirmed through this study. Further research using a more diverse combination of optimized wireless elements and HPM structures is warranted. Therefore, we plan to examine a more optimized combination of ‘multi-channel RF coils compared with a birdcage wireless element (BCWE) and *sc*HPM’ and ‘BP-BC RF coils with MCWE and *sc*HPM’ in future studies.

## Figures and Tables

**Table 1:** The maximum values of EM-field simulation results (|$B_{1}$|-, |$B_{1}^{+}$|-, |$B_{1}^{-}$|-, and |E|-field) using oil-based cylindrical phantom and human head model.

		BP-BC RF Coil Alone	BP-BC RF Coil + *hc*HPM	BP-BC RF Coil + *sc*HPM	
		Center-point values of |$B_{1}$|-field			×10^−5^ [μT]
Oil-based cylindrical phantom	*wo*-MCWE	0.349	0.292	0.383	
	*w*-MCWE	0.402	0.304	0.531	
Human head model	*wo*-MCWE	0.787	0.675	0.807	
	*w*-MCWE	0.853	0.717	1.042	
		$\mathrm{SD}\;\mathrm{values}\;\mathrm{of}\;|B_{1}$|-field			×10^−5^ [μT]
Oil-based cylindrical phantom	*wo*-MCWE	0.004	0.054	0.075	
	*w*-MCWE	0.003	0.051	0.019	
Human head model	*wo*-MCWE	0.110	0.111	0.113	
	*w*-MCWE	0.121	0.116	0.1477	
		$\mathrm{Max}\;\mathrm{values}\;\mathrm{of}\;|B_{1}^{+}$|-field			×10^−5^ [μT]
Oil-based cylindrical phantom	*wo*-MCWE	0.247	0.207	0.291	
	*w*-MCWE	0.284	0.215	0.443	
Human head model	*wo*-MCWE	0.588	0.491	0.609	
	*w*-MCWE	0.637	0.522	0.7808	
		$\mathrm{SD}\;\mathrm{values}\;\mathrm{of}\;|B_{1}^{+}$|-field			×10^−5^ [μT]
Oil-based cylindrical phantom	*wo*-MCWE	0.004	0.044	0.004	
	*w*-MCWE	0.003	0.039	0.011	
Human head model	*wo*-MCWE	0.093	0.089	0.094	
	*w*-MCWE	0.100	0.092	0.120	
		$\mathrm{Max}\;\mathrm{values}\;\mathrm{of}\;|B_{1}^{-}$|-field			×10^−5^ [μT]
Oil-based cylindrical phantom	*wo*-MCWE	0.050	0.106	0.071	
	*w*-MCWE	0.058	0.083	0.128	
Human head model	*wo*-MCWE	0.271	0.237	0.273	
	*w*-MCWE	0.292	0.247	0.346	
		$\mathrm{SD}\;\mathrm{values}\;\mathrm{of}\;|B_{1}^{-}$|-field			×10^−5^ [μT]
Oil-based cylindrical phantom	*wo*-MCWE	0.014	0.017	0.016	
	*w*-MCWE	0.014	0.012	0.025	
Human head model	*wo*-MCWE	0.048	0.044	0.049	
	*w*-MCWE	0.052	0.045	0.060	
		Max values of |E|-field			[V/m]
Oil-based cylindrical phantom	*wo*-MCWE	260.257	251.539	202.869	
	*w*-MCWE	224.388	246.640	296.424	
Human head model	*wo*-MCWE	727.420	567.106	711.303	
	*w*-MCWE	740.012	586.246	918.380	

**Table 2:** Norm-COEF (Normalized coefficient), whole average SAR, and max SAR values of EM-field simulation.

	BP-BC RF Coil Alone	BP-BC RF Coil + *hc*HPM	BP-BC RF Coil + *sc*HPM	
	Norm-COEF (Normalized Coefficient)			[a.u.]
*wo*-MCWE	0.362	0.431	0.352	
*w*-MCWE	0.334	0.406	0.273	
	Whole average SAR (Mean SAR values)			[W/Kg]
*wo*-MCWE	0.209	0.245	0.215	
*w*-MCWE	0.216	0.235	0.211	
	Max SAR values			[W/Kg]
*wo*-MCWE	8.203	12.574	8.461	
*w*-MCWE	8.376	11.662	8.248	

## Data Availability

Not applicable.
